# Urogenital Tuberculosis and Delayed Diagnosis: A Qualitative Study

**DOI:** 10.5152/tud.2024.24028

**Published:** 2024-05-01

**Authors:** Augusto de Azevedo Barreto, Humberto Elias Lopes, José Murillo Bastos Netto, André Avarese Figueiredo

**Affiliations:** 1 Núcleo Interdisciplinar de Pesquisa em Urologia (NIPU), Federal University of Juiz de Fora, Minas Gerais, Brazil; 2 Department of Surgery, Federal University of Juiz de Fora, Minas Gerais, Brazil; 3 University Hospital—Federal University of Juiz de Fora, Minas Gerais, Brazil

**Keywords:** Urogenital tuberculosis,, qualitative research,, diagnosis,, urinary bladder,, codes

## Abstract

**Objectives:**

To identify the causes of delayed diagnosis of urogenital tuberculosis (UGT) through a qualitative study of patients with contracted bladder due to UGT.

**Materials and Methods:**

Eight patients diagnosed with contracted bladder due to UGT were evaluated. Data were obtained using face-to-face in-depth interviews and supplemented with medical records analysis and personal medical files. The identification of situations of diagnosis delay was coded by 2 urologists after data analyses. Codes were divided into 3 categories related to its causes: (1) health system; (2) disease factors; and (3) medical factors.

**Results:**

The 8 interviews produced 220 minutes of audio and 1.3 GB of scanned documents. The most frequent categories were “Medical factors,” followed by “Disease factors” and “Health system.” The codes “No clinical-laboratory-radiological suspicion” and “No clinical suspicion” were the most frequent, both belonging to “Medical factors.” Clinically, tuberculosis simulates other pathologies and lacks specific tests with adequate sensitivity. The low representation of “Health system” codes indicates that access to public and private health services does not influence delayed diagnosis. The lack of clinical and radiological suspicion and the lack of knowledge of UGT features are the main reasons for diagnosis delay.

**Conclusions:**

The causes of delayed diagnosis in our sample were related to “Medical factors,” followed by “Disease factors.” Better understanding UGT features is an important topic in continuous medical education.

**Main Points**

- UGT is a progressive disease, and early diagnosis is essential to avoid serious urinary tract injuries.
- In this study, delayed diagnosis is due to medical and disease factors.
- Correct propaedeutics and symptom identification, together with unfamiliarity regarding low-sensitivity TB tests, rests as major causes of delayed diagnosis.

## Introduction

Tuberculosis is an airborne infectious disease caused by *Mycobacterium tuberculosis* (*Mtb*) that mainly affects low-income, inadequate nutrition and immunosuppressed populations. The pulmonary form of the disease is the most common; however, extrapulmonary tuberculosis can affect 16% of patients.^1^ Urogenital tuberculosis (UGT) is the second or sometimes the third most frequent presentation of extrapulmonary disease and occurs after hematogenous dissemination of the bacillus from the pulmonary focus.^2^ Urogenital tuberculosis becomes active after a long latency period and activation usually occurs in only one of the kidneys. The inflammatory process begins with a granulomatous reaction, and renal disease evolution is slow but progressive, disrupting the entire renal architecture and forming areas of caseous necrosis. The invasion of the collecting system starts when papillary granulomas rupture, initiating the urinary dissemination of the bacillus, with involvement of the ipsilateral ureter and the urinary bladder. Bladder tuberculosis can be classified into 4 stages: stage 1 (tubercle-infiltrative bladder TB); stage 2 (erosive-ulcerous bladder TB); stage 3 (interstitial cystitis/painful bladder syndrome); and stage 4 (contracted or “thimble bladder” up to full obliteration).^3^ At stage 4, the progressive fibrosis of the bladder wall causes low bladder compliance, distortion of the ureterovesical junction, and secondary reflux, with transmission of high bladder pressure to the upper urinary tract, which may damage kidney function.^4^ Since UGT has a progressive and sequential evolution, patients with contracted bladder due to UGT represent advanced disease and therefore a delayed diagnosis.

Understanding the pathophysiology of UGT reinforces the need for early diagnosis and prescription of first-line TB drugs in the early stages of the disease. Even though UGT is known as “the great imitator,”^5^ there are clinical clues (signs and symptoms) together with radiology and laboratory findings that could support earlier diagnosis.^6^ Understanding and describing the causes of diagnostic delay can lead to proper actions in many knowledge fields.

In this way, qualitative studies differ from quantitative ones regarding the main research question. Once it is: “What” are the causes of diagnosis delay, qualitative study is the right tool to go deep in a row of patient’s history and medical records, performing detailed analysis of each medical history to find the information needed.^7,8^ The objective of this study was to identify the causes of delayed diagnosis through a qualitative study of patients with “contracted bladder” due to UGT.

## Materials and Methods

This study followed the guidelines of the Consolidated Criteria for Reporting Qualitative Research (COREQ).

A qualitative study—subtype content analysis—was conducted through semi-structured face-to-face in-depth interviews and the collection of personal and institutional medical records. The researcher responsible for data collection was a general urologist who graduated in 1996, with 20 years’ experience both the public and private sectors, as well as inpatient and outpatient units. The researcher did not know any of the individuals in the sample at the time of recruitment and vice versa.

The sampling strategy used was “criterion sampling.”^9^ All patients diagnosed with UGT and contracted bladder in our database in the last 10 years were recruited.

The inclusion criteria were patients with UGT confirmed through bacteriological or histological diagnosis and the presence of a contracted bladder. The exclusion criteria were the inability to attend the interview in person or refusal to sign the informed consent. There were no losses after recruitment.

Delayed diagnosis was defined as the presence of a contracted bladder at the moment of the definitive diagnosis of urogenital tuberculosis.

Contracted bladder was defined as a thickened bladder with a capacity of less than 100 mL in ultrasonography or urethrocystography measures. The study was approved by the University Hospital - Federal University of Juiz de Fora Research Ethics Committee under number 4 418 369. Interviews were conducted in 2020 and 2021 in the outpatient unit of the University Hospital - Juiz de Fora, Minas Gerais - Brazil. Informed consent was obtained from all individual participants included in the study.

The interview began with the question: “Please describe in detail how the diagnosis of tuberculosis occurred, starting from the day you were feeling well with no symptoms.” Subsequently, the interviews were conducted in such a way that the patient and family could report in detail the clinical evolution and the experiences between the onset of symptoms and the diagnosis of UGT. Each audio interview was recorded and transcribed.

Two authors read the transcripts separately as well as medical records and performed the coding process using ATLAS.ti software version 9. Coding consisted of carefully reading the transcriptions several times and analyzing medical records such as laboratory, imaging, histological, and urodynamic tests. The purpose was highlighting situations related to delayed diagnosis both in medical records and semi-structured interviews. Subsequently, each interview was reviewed, and all situations associated with diagnosis delay were quoted and had a code applied. Codes were numbered and classified into 3 categories according to the general factor of delay: Category 1—Health system factors; Category 2—Disease factors; and Category 3—Medical factors.

## Results

The study sample consisted of 8 patients, i.e., 6 men and 2 women aged between 30 and 72 years. There were no losses after recruitment. “Diagnosis of UGT was confirmed through bacteriology in 2 patients and through histology in the remaining 6 patients: 1 from bladder biopsy and 5 from kidney histology after pathology findings in nephrectomy specimens.” All individuals had contracted bladder, and 7 had a non-functional unilateral hydronephrotic kidney. All participants were indicated for bladder augmentation, which was performed with detubularized ileum in 3 patients and detubularized sigmoid colon in 4 others. One patient has not yet undergone surgery.

The 8 interviews generated 3 hours and 40 minutes of audio recordings, with an average time of 24 minutes per interview. The total volume of digitized data was 1.30 GB. Transcribed interviews produced 56 quotations related to situations of diagnostic delay, and 26 codes were applied. Each code received a number identification (ID) and was grouped into one of the 3 categories.

### Category 1—Health System

Codes 01–05 are related to the Health system (Table 1). Patients and families reported difficulty accessing specialists, laboratory tests, and basic care. The unavailability of tests in the health system was also identified in one of the interviews.

### Category 2—Disease Factors

Codes 06-17 are related to disease features (Table 1). The interviews revealed the ability of the disease to mimic other pathologies, such as benign prostatic hyperplasia, overactive bladder, lithiasis, pyelonephritis, and prostatitis. The documentary analysis, along with interviews, identified synchronous urinary lithiasis in 3 patients and an extensive set of situations in which histological exams, urine culture for *Mtb,* and smear microscopy for acid-fast bacilli (AFB) resulted in false negatives. Specimens from transurethral resection of the prostate and prostate biopsy, as well as nephrectomy specimens, were negative for direct microscopy identification of AFB or did not show typical granulomatous inflammation. In one patient, the disease mimicked a malignant neoplasm.

### Category 3—Medical Factors

Codes 18-26 are related to iatrogenic factors of diagnosis delay (Table 1). There were situations in which the physician did not clinically suspect UGT even with signs and symptoms such as persistent hematuria and unilateral low back pain. Findings such as sterile pyuria and hydronephrosis without an apparent cause also did not raise suspicion among clinicians and specialists. Physicians conducted no further investigation in the presence of *Mtb* negative cultures, demonstrating full confidence in the method despite its low sensitivity. In this regard, even for a patient with a contracted bladder, urologists failed to diagnose UGT if the urine culture was negative. The absence of references to suspected tuberculosis in pathology reports was also identified in the data. In some cases, a negative smear microscopy deceived urologists and made clinical UGT diagnosis impossible.

Table 2 summarizes the magnitude (frequency) of the codes. The preponderance of Category 3—Medical factors—allows us to conclude that factors causing diagnostic delay are related to insufficient clinical and radiological suspicion and a lack of knowledge on UGT features.

Table 3 shows examples of phrases that were coded and rest as representative comments for each thematic category.

The data show that the knowledge of some UGT features could lead to an earlier diagnosis, preventing destruction of the urinary tract:

1. Urogenital tuberculosis should be suspected in the presence of symptoms like hematuria, pollakiuria, and lumbar pain associated with sterile pyuria and radiological findings of thickening/obstruction of the urinary system without an apparent cause.
2. *Mycobacterium tuberculosis* urine culture should include at least 3 clinical samples on different days, and given its low sensitivity, negative results do not exclude UGT.
3. The presence of granulomatous inflammation in the kidney, prostate, bladder, and epididymis allows the diagnosis of tuberculosis, even in the absence of the bacillus.
4. The absence of granulomatous inflammation in the kidney and prostate does not exclude the diagnosis of tuberculosis.

Summarizing, UGT can be diagnosed, even with negative bacteriological/histological tests, if hematuria and/or pollakiuria along with typical radiological findings are present. Radiological findings are mainly:

Unilateral dilation/thickening of the renal excretory route without an apparent cause.

Contracted bladder with unilateral or bilateral hydronephrosis.

## Discussion

The diagnosis of UGT remains a challenge for urologists due to nonspecific symptoms and the small number of bacilli in urine. Even with all the technological advances, little progress has been made toward early diagnosis. Most forms of EPTB are not transmissible and, therefore, do not represent a risk to public health as seen in the small amount of information on EPTB in the WHO annual report on tuberculosis.^10^ This is the present situation of UGT.

The pathophysiology of UGT described in the literature^4^ suggests a sequential and progressive evolution, starting with unilateral kidney tuberculosis, stenosis of the urinary tract, bladder tuberculosis (thimble bladder), and contralateral reflux. Clearly, diagnosis in the early stage of the disease and the prescription of first-line anti-TB drugs interrupts the natural sequence of UGT, preserving the urinary system. According to Nakane et al,^11^ the delay can be described into 2 stages: the first is the period between symptom onset and the initial visit to the doctor (T1) and the second comprises the period between the medical consultation and diagnosis (T2). In our sample, the T1 value was 1 month in all patients, and mean T2 was 37 months (ranging from 6 to 69 months). Obtaining detailed information on the trajectory of physicians and patients from the first consultation until the diagnosis of UGT provided the motivation for our study, and because the variables were unknown, we chose qualitative rather than quantitative methodology to understand the phenomenon.

The qualitative data indicated that medical factors were the most important in the delayed diagnosis of UGT, followed by factors related to the disease and then those related to the Health system. Doctors had the chance for an early syndromic diagnosis in many situations. In most individuals, the disease evolved in a natural and predictable way.^4^ Clinically, the time when a syndromic diagnosis was possible was observed on 7 occasions in the qualitative data (Code 19—No clinical laboratory and radiological suspicion). This code was applied whenever there was the presence of a symptom (hematuria, pollakiuria, lumbar pain, pelvic pain, and scrotal pain) associated with sterile pyuria and/or hydronephrosis, and the doctor did not mention UGT. The difficulty of physicians in relating this syndrome to UGT is a relevant issue. It may be caused by a lack of knowledge of UGT’s natural history and its low prevalence.

Pathology reports also contributed to diagnostic delays. Pathologists observed caseous granuloma and did not report UGT as the main diagnostic hypothesis. Although granulomatous inflammatory response is not exclusive to *Mtb* infection, this information is important to accurate diagnosis. Multiple stimuli lead to granulomas, such as infectious agents, foreign bodies, and other inflammatory/autoimmune diseases, not to say bacteria, fungi, protozoa, and viruses. Schistosomiasis and syphilis are known as granulomatous diseases. Among autoimmune disorders, sarcoidosis and Crohn’s are the most frequent pathologies causing granulomas.^12^ Prevalence of TB in humans vastly exceeds all other granulomatous diseases, and therefore, the role of the pathologist becomes essential in early diagnosis.^9^

In the medical dimension, the attempt to isolate *MTB* clearly led to diagnostic delays. We observed situations where physicians accepted a negative AFB microscopy in the anatomical pathological report as absolute truth and promptly disregarded UGT. We also noted requests for an *Mtb* culture and/or AFB smear microscopy in one and only one urine sample. Once negative, medical staff looked away towards other pathologies, withdrawing UGT as a possible diagnosis.

Detection of *Mtb* is not possible in all cases of UGT and is not an easy task.^3^ Currently, there are many tests to identify the bacillus. Smear microscopy using Ziehl–Neelsen (ZN) or auramine staining in sputum, pus, discharge, tissues, and urine has been available for over 50 years but has its drawbacks. Culture in Lowenstein–Jensen medium remains the gold standard test for UGT. There are also faster automated liquid culture systems available for *Mtb* detection (Becton Dickinson BACTEC MGIT 960) and DNA sequencing technologies. DNA sequencing methods, such as the GeneXpert MTB/RIF Assay andHain MTBDR Plus line probe assay*,* providereliable molecular diagnostics using tissue or urine samples and are being used globally to screen for *Mtb* and the most prevalent genetic mutations associated with resistance to isoniazid and/or rifampin.^3,13,14^

Recently, Pyrosequencing (PSQ) has emerged as a real-time assay for rapid sequencing of small segments of genomic DNA to detect multidrug and extensively drug-resistant tuberculosis (M/XDR-TB) accurately and reliably. It can overcome the limitations of previous M/XDR-TB assays and detect resistance to more drugs in a shorter time. It not only determines the presence or absence of these mutations but also displays the exact sequence data to guide treatment decisions. Compared to GeneXpert, PSQ can detect resistance to more drugs, and compared to the GenoType MTBDR method, it is more accurate and easier to handle.^14^

Other reasons for delays in diagnosis are related to the nonspecific clinical presentation of UGT. In 2015, Kulchavenya et al^15^described the ability of tuberculosis to simulate other urinary pathologies and escape early diagnosis. In our data, UGT simulated common bacterial pyelonephritis, cystitis, prostatitis, and benign prostatic hyperplasia. The synchronous presence of urinary lithiasis was also an important reason for delayed diagnosis in patients with UGT. Clinical diagnosis of patients with urinary lithiasis and UGT is invariably delayed, and unlikely if both pathologies occur simultaneously. We observed situations in which a history of urinary stones justified findings such as sterile pyuria or hematuria and thus delayed the diagnosis of UGT.

Given our results, UGT should be included in continuing medical education programs to reduce “T2” (time between the medical visit and diagnosis).^11^ The low sensitivity of diagnostic methods (smear microscopy for AFB, *Mtb* solid and liquid culture, and molecular assays) should be widely known by physicians. There is urgency in defining radiological criteria for urologist prescription of first-line anti-TB drugs without a proven etiological diagnosis of the disease.

Our findings allow us to conclude that the causes of delayed diagnosis in our sample were mainly related to “Medical factors,” followed by “Disease factors.” Clinically, UGT simulates other highly prevalent pathologies and lacks specific tests with adequate sensitivity. A better understanding of UGT features is an important topic in continuous medical education.

## Figures and Tables

**Table 1.** Emerging Codes After Interviews and Content Analyses

ID	Code
01	Difficulty in accessing basic care
02	Difficulty in accessing specialists
03	Difficulty accessing laboratory tests
04	Difficulty in accessing imaging tests
05	Unavailability of tests in the health system
06	Synchronic lithiasis present
07	Simulates BPH^1^
08	Simulates overactive bladder
09	Simulates lithiasis
10	Simulates bacterial pyelonephritis
11	Simulates bacterial cystitis
12	Simulates bacterial prostatitis
13	False negative culture and/or AFB^2^—urine
14	Inconclusive pathology findings—prostate biopsy
15	False negative AFB microscopy—tissue sample—prostate
16	Inconclusive pathology findings—nephrectomy
17	Simulates a malignant neoplasm
18	No clinical suspicion
19	No clinical laboratory and radiological suspicion
20	No suspicion in the presence of contracted bladder
21	Request for culture and/or AFB for only one urine sample
22	Admitting false negative results for cultures and/or AFB microscopy
23	Disregarding pathology report of “granulomatous inflammation” as TB^3^
24	Accepting a negative AFB microscopy as thuth
25	Absence of reference to TB as suspected by the pathologist
26	Absence of reference to TB as a diagnostic hypothesis by the pathologist

^1^BPH, benign prostatic hyperplasia; ^2^AFB, acid-fast bacill; ^3^TB, tuberculosis.

**Table 2.** Codes Magnitude

ID	Code	Magnitude	Category
19	No clinical laboratory and radiological suspicion	7	Medical factors
18	No clinical suspicion	5	Medical factors
10	Simulates bacterial pyelonephritis	4	Disease factors
26	Absence of reference to TB^1^ as a diagnostic hypothesis by the pathologist		Medical factors
24	Accepting a negative AFB^2^ microscopy as thuth	3	Medical factors
11	Simulates bacterial cystitis	3	Disease factors
23	Disregarding pathology report of “granulomatous inflammation” as TB	3	Medical factors
06	Synchronic lithiasis present	3	Disease factors
02	Difficulty in accessing specialists	2	Health system
20	Request for culture and/or AFB for only one urine sample	2	Medical factors
03	Difficulty accessing laboratory tests	2	Health system
08	Simulates overactive bladder	2	Disease factors
25	Absence of reference to TB as suspected by the pathologist	2	Medical factors
12	Simulates bacterial prostatitis	2	Disease factors
13	False negative culture and/or AFB^2^—urine	**2 **	Disease factors

^1^TB, tuberculosis; ^2^AFB, acid-fast bacilli.

**Table 3.** Representative References by Categories

Category 1—Health system
“The first time I took too long to make an appointment at the health center.” “When you come to ask (about the exam) they say, “No, we lost the results here. You’ll have to get another one.”
Category 2—Disease factors
“Then, the fever wouldn’t go down; he was hospitalized for 22 days, only changing antibiotics.”
“She started feeling the pain she said she had and was feeling sick. She went to the hospital, and they treated it as a urinary tract infection.”
“When I urinated, I spent 10 minutes, and then, I felt like I had to go again within 10, 15, to 20 minutes. Then, I started to get a fever.”
Category 3—Medical factors
“He underwent surgery to lift the bladder and widen the canal because according to him, the bladder was low and needed to be lifted.”
“So, he thought that perhaps he was urinating a lot because of the enlarged prostate size compressing his bladder.”
“From the AFB culture? It didn’t get done, or I think it was done only on 1 day, and it was negative. It has already ruled out the hypothesis.”

Source: Prepared by the author—2023.
